# Impact of isovalent and aliovalent substitution on the mechanical and thermal properties of Gd_2_Zr_2_O_7_

**DOI:** 10.1038/s41598-017-06725-8

**Published:** 2017-07-25

**Authors:** S. Zhang, H. B. Zhang, F. A. Zhao, M. Jiang, H. Y. Xiao, Z. J. Liu, X. T. Zu

**Affiliations:** 1 School of Physical Electronics, University of Electronic Science and Technology of China, Chengdu, 610054 China; 2 Institute of Nuclear Physics and Chemistry, Chinese Academy of Engineering Physics, Mianyang, 621900 China; 3 Institute of Fundamental and Frontier Sciences, University of Electronic and Technology of China, Chengdu, 610054 China; 4 Department of Physics, Physics, Lanzhou City University, Lanzhou, 730070 China

## Abstract

In this study, a density functional theory method is employed to investigate the effects of isovalent and aliovalent substitution of Sm^3+^ on the phase stability, thermo-physical properties and electronic structure of Gd_2_Zr_2_O_7_. It is shown that the isovalent substitution of Sm^3+^ for Gd^3+^ results in the formation of Gd_2_Zr_2_O_7_-Sm_2_Zr_2_O_7_ solid solution, which retains the pyrochlore structure and has slight effects on the elastic moduli, ductility, Debye temperature and band gap of Gd_2_Zr_2_O_7_. As for the aliovalent substitution of Sm^3+^ for Zr^4+^ site, a pyrochlore-to-defect fluorite structural transition is induced, and the mechanical, thermal properties and electronic structures are influenced significantly. As compared with the Gd_2_Zr_2_O_7_, the resulted Gd_2_Sm_y_Zr_2-y_O_7_ compositions have much smaller elastic moduli, better ductility and smaller Debye temperature. Especially, an amount of electrons distribute on the fermi level and they are expected to have larger thermal conductivity than Gd_2_Zr_2_O_7_. This study suggests an alternative way to engineer the thermo-physical properties of Gd_2_Zr_2_O_7_ and will be beneficial for its applications under stress and high temperature.

## Introduction

The rare-earth zirconates, with chemical formula A_2_Zr_2_O_7_ (A = Y or another rare earth elements)^1, 2^, exhibit ordered pyrochlore-type structure or defect fluorite-type structure, which is mainly governed by the ionic radii of A^3+^ and Zr^4+^ ^3^. They have attracted the attention of many researchers, due to their good chemical and mechanical stability, excellent catalytic activity, high ionic conductivity, ferromagnetism, luminescence as well as strong resistance to amorphization under irradiation^4–8^. Owing to these outstanding properties, the rare-earth zirconates have a wide range of technical applications, e.g., ceramic thermal barrier coating^9, 10^, oxidation catalyst^5, 11^, solid electrolyte^12^, hosts of actinides in nuclear waste^13^ and oxygen gas sensor^14^.

Of the zirconate pyrochlores, Gd_2_Zr_2_O_7_ is of special interest due to its good thermo-physical properties^15–19^. Shimamura *et al*. have measured the thermal expansion of a series of zirconate pyrochlores employing high-temperature X-ray diffraction and found that the thermal expansion coefficient of Gd_2_Zr_2_O_7_ is larger than other zirconates during the temperature range of 400–1600 °C^20^. The thermal conductivity of rare-earth zirconates has been investigated by Wang *et al*.^21^, who reported that the thermal conductivity of 1.15–1.43 W/mK for Gd_2_Zr_2_O_7_ is lower than that of Yb_2_Zr_2_O_7_, Dy_2_Zr_2_O_7_ and 7YSZ between 25 °C and 800 °C. In recent years, a great number of investigations have been carried out on substitution of lanthanides for Gd-site to engineer the thermo-physical properties of Gd_2_Zr_2_O_7_^22–25^. Guo *et al*. synthesized (Gd_1-x_Yb_x_)_2_Zr_2_O_7_ (x = 0, 0.1, 0.3, 0.5, 0.7) using solid state reaction and suggested that the thermal conductivity of 0.88–1.00 W/mk for (Gd_1-x_Yb_x_)_2_Zr_2_O_7_ is about 20% lower than that of Gd_2_Zr_2_O_7_ (1.18 W/mk) at 1400 °C^26^. Wan *et al*. predicted that, among the (La_x_Gd_1-x_)_2_Zr_2_O_7_ (0 ≤ x ≤ 1) systems, the LaGdZr_2_O_7_ has the minimum thermal conductivity, which is about 20–25% lower than that of Gd_2_Zr_2_O_7_^18^. In recent years, both Liu *et al*.^19^. and Pan *et al*.^23^ reported that the thermal diffusivity of (Sm_x_Gd_1-x_)_2_Zr_2_O_7_ (0 ≤ x ≤ 1) are lower than those of pure Gd_2_Zr_2_O_7_ and Sm_2_Zr_2_O_7_. Especially, Sm_2_Zr_2_O_7_-Gd_2_Zr_2_O_7_ solid solutions have lower Young’s modulus than (La_x_Gd_1-x_)_2_Zr_2_O_7_ (0 < x < 1) at room temperature and larger thermal expansion coefficients than (Gd_1-x_Yb_x_)_2_Zr_2_O_7_ (x = 0, 0.1, 0.3, 0.5, 0.7) from 300 °C to 900 °C^18, 19, 23, 26^. These investigations are mainly experimental studies, and the related theoretical investigations are relatively much fewer^27, 28^.

Very recently, the Th^4+^ ion incorporation into Gd^3+^ and Zr^4+^ sites in Gd_2_Zr_2_O_7_ was investigated by first-principles calculations^28^. Unexpectedly, the aliovalent substitution of Th^4+^ for Gd^3+^ turns out to be thermodynamically stable and such substitution even results in better thermo-physical properties than the pure Gd_2_Zr_2_O_7_^28^. This thus arouses our interest that whether the aliovalent substitution of Ln^3+^ (Ln = lanthanide elements) for Zr^4+^ sites are energetically and mechanically stable or not? If yes, will the substitution of Ln^3+^ for Zr^4+^ sites cause different thermo-mechanical properties from the isovalent substitution of Ln^3+^ for Gd^3+^ sites? In this study, we choose Sm^3+^ as a model and investigate the phase stability and thermo-physical properties of Gd_2_Zr_2_O_7_ with isovalent and aliovalent substitution of Ln^3+^ for Gd^3+^ and Zr^4+^ sites by employing the density functional theory (DFT) method. It reveals that the Sm_y_Gd_2-y_Zr_2_O_7_ retains the pyrochlore structure and the isovalent substitution of Sm^3+^ for Gd^3+^ sites influences slightly the mechanical and thermal properties of Gd_2_Zr_2_O_7_. On the other hand, the aliovalent substitution of Sm^3+^ for Zr^4+^ sites induces pyrochlore-to-fluorite structural transition and affects significantly the elastic moduli, Debye temperature and thermal conductivity. The presented results provide a new way to tune the thermo-physical properties of Gd_2_Zr_2_O_7_ and will have important implications in advancing the further related experimental and theoretical studies for its applications under high temperature.

## Results and Discussion

### Structural stability of Sm_y_Gd_2-y_Zr_2_O_7_ and Gd_2_Sm_y_Zr_2-y_O_7_ (0 ≤ y ≤ 2)

**Figure 1:**
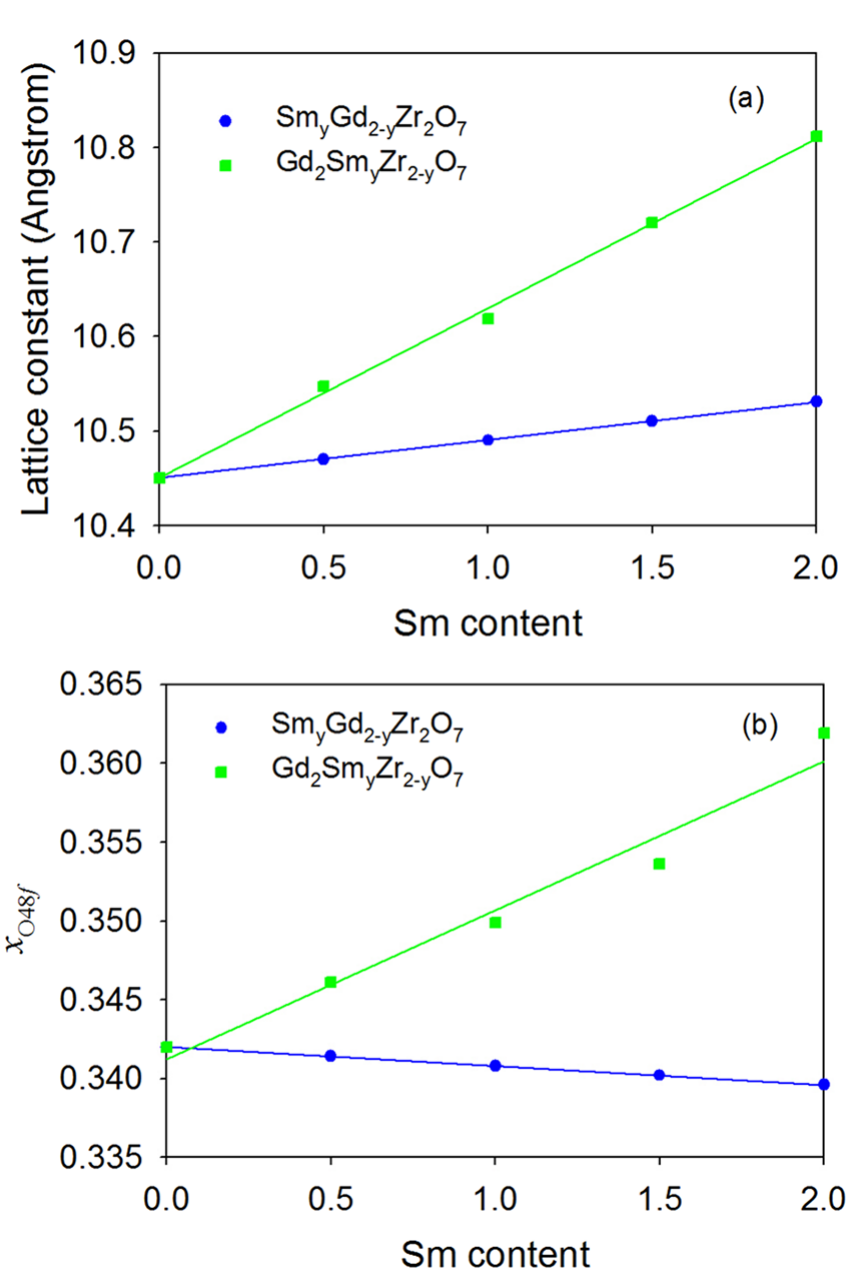
Variation of (**a**) lattice constants and (**b**) positional parameter *x*_O48*f*_ for Sm_y_Gd_2-y_Zr_2_O_7_ and Gd_2_Sm_y_Zr_2-y_O_7_ (0 ≤ y ≤ 2) as a function of Sm content.

**Figure 2:**
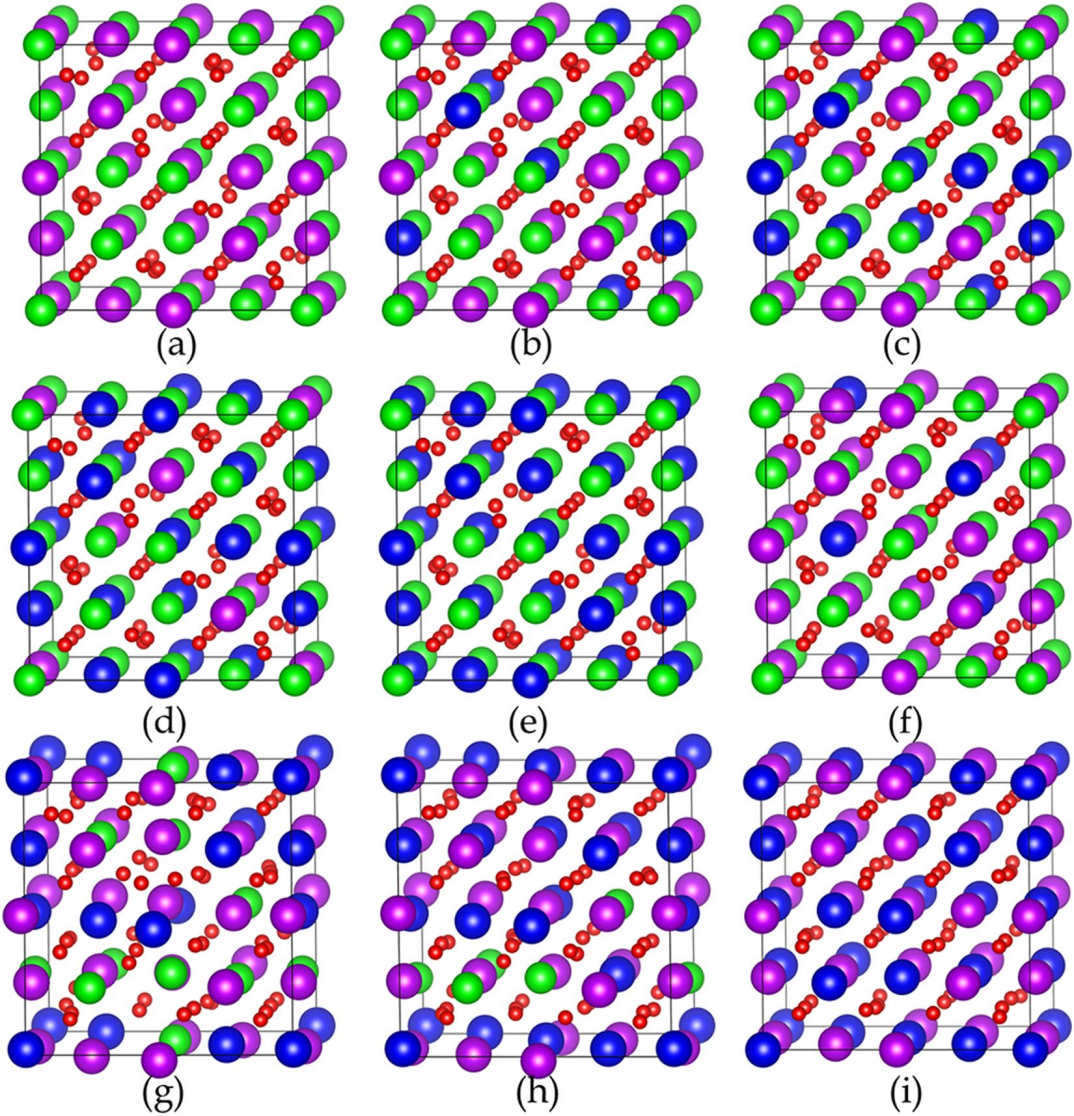
Schematic view of the optimized configurations for (**a**) Gd_2_Zr_2_O_7_, (**b**) Sm_0.5_Gd_1.5_Zr_2_O_7_, (**c**) SmGdZr_2_O_7_, (**d**) Sm_1.5_Gd_0.5_Zr_2_O_7_, (**e**) Sm_2_Zr_2_O_7_, (**f**) Gd_2_Sm_0.5_Zr_1.5_O_7_, (**g**) Gd_2_SmZrO_7_, (**h**) Gd_2_Sm_1.5_Zr_0.5_O_7_, (**i**) Gd_2_Sm_2_O_7_. The blue, purple, green and red spheres represent Sm, Gd, Zr and O atoms, respectively.

In this study, Sm substitution for both Gd-site and Zr-site in Gd_2_Zr_2_O_7_ with different concentrations are considered, resulting in Sm_y_Gd_2-y_Zr_2_O_7_ and Gd_2_Sm_y_Zr_2-y_O_7_ (y = 0, 0.5, 1, 1.5, 2). The geometrical structures of Sm_y_Gd_2-y_Zr_2_O_7_ and Gd_2_Sm_y_Zr_2-y_O_7_ are optimized firstly. In order to test whether local density approximation (LDA)^29^ or generalized gradient approximation (GGA)^30^ is more appropriate to describe the exchange-correlation interaction between electrons, both LDA and GGA methods are employed to relax the structures of Gd_2_Zr_2_O_7_ and Sm_2_Zr_2_O_7_. For Gd_2_Zr_2_O_7_, the lattice constant *a*_*0*_ and *x*_O48*f*_ obtained by LDA are 10.451 Å and 0.342, respectively, agreeing well with the experimental values of *a*_*0*_ = 10.472 Å and *x*_O48*f*_ = 0.345^31, 32^. As compared with the LDA results, the values of *a*_*0*_ = 10.641 Å and *x*_O48*f*_ = 0.339 calculated by GGA deviate much more from the experimental results. As for Sm_2_Zr_2_O_7_, the LDA calculations yield a lattice constant of 10.531 Å and an *x*_O48*f*_ parameter of 0.34, which are also comparable with the experimental data of *a*_*0*_ = 10.514 Å and *x*_O48*f*_ = 0.342^31, 32^. On the other hand, the GGA results of *a*_*0*_ = 10.715 Å and *x*_O48*f*_ = 0.337 are in bad agreement with the experimental measurement. Obviously, the structural parameters obtained by LDA are in better agreement with experiments than the GGA method. The LDA method, thus, is employed in all the subsequent calculations. The calculated lattice constant *a*_*0*_ and *x*_O48*f*_ for Sm_y_Gd_2-y_Zr_2_O_7_ and Gd_2_Sm_y_Zr_2-y_O_7_ as a function of Sm content are plotted in Fig. 1a and b, respectively. As Sm substitutes for Gd-site in Gd_2_Zr_2_O_7_, it is found that the resulted Sm_y_Gd_2-y_Zr_2_O_7_ compositions remain the ideal pyrochlore structure. Especially, the lattice constant *a*_*0*_ and the O_48*f*_ positional parameter  *x*_O48*f*_ increase and decrease linearly with the increasing Sm concentration, suggesting that he lattice parameters of Sm_y_Gd_2-y_Zr_2_O_7_ follow well the Vegard’s law, i.e., $a({{\rm{Sm}}}_{{\rm{y}}}{{\rm{Gd}}}_{{\rm{2}}-{\rm{y}}}{{\rm{Zr}}}_{{\rm{2}}}{{\rm{O}}}_{{\rm{7}}})=\frac{2-y}{2}\times a({{\rm{Gd}}}_{{\rm{2}}}{{\rm{Zr}}}_{{\rm{2}}}{{\rm{O}}}_{{\rm{7}}})+\frac{y}{2}\times a({{\rm{Sm}}}_{{\rm{2}}}{{\rm{Zr}}}_{{\rm{2}}}{{\rm{O}}}_{{\rm{7}}})$. These results indicate that the Gd_2_Zr_2_O_7_-Sm_2_Zr_2_O_7_ solid solution is formed by Sm substitution into Gd-site in Gd_2_Zr_2_O_7_, agreeing well with the experimental observation^19^. Experimentally, Liu *et al*. also found that the lattice constants increase linearly with different compositions for (Sm_x_Gd_1−x_)_2_Zr_2_O_7_ system from x = 0 (Gd_2_Zr_2_O_7_) to x = 1.0 (Sm_2_Zr_2_O_7_), and the Gd_2_Zr_2_O_7_ and Sm_2_Zr_2_O_7_ ceramics are infinitely solid solution^19^. As Sm substitutes for Zr-site, i.e. Gd_2_Sm_y_Zr_2-y_O_7_, the lattice constants increase more significantly than the case of Sm_y_Gd_2-y_Zr_2_O_7_ and there is a small deviation from the Vegard’s law. It is noticeable that the *x*_O48*f*_ of Gd_2_Sm_y_Zr_2-y_O_7_ increases remarkably with the increasing Sm content instead of decreasing as the case of Sm_y_Gd_2-y_Zr_2_O_7_. In A_2_B_2_O_7_ pyrochlores, the *x*_O48*f*_ positional parameter can be used to describe the degree of distortion of < B-O > octahedron and it is located within the range of 0.3125 to 0.375. Generally, the material with *x*_O48*f*_ closer to 0.3125 has the ordered pyrochlore structure and the one with *x*_O48*f*_ closer to 0.375 is more likely to undergo a transition from pyrochlore to defect-fluorite structure^31, 33–35^. Our calculations show that the *x*_O48*f*_ for Gd_2_Sm_y_Zr_2-y_O_7_ with high content of Sm approaches to be 0.375, implying that order-disorder transition may occur in the systems. The schematic view of optimized configurations of Sm_y_Gd_2-y_Zr_2_O_7_ and Gd_2_Sm_y_Zr_2-y_O_7_ is illustrated in Fig. 2. As can be seen in the figure, Sm_y_Gd_2-y_Zr_2_O_7_ still maintains the pyrochlore-type structure, while Gd_2_Sm_y_Zr_2-y_O_7_ exhibits defective fluorite-type structure due to the significant disordering of the anions. These results suggest that Sm substitution for Gd-site has minor effects on the pyrochlore structure of Gd_2_Zr_2_O_7_, as evidenced by the small changes in the < Gd-O > and < Zr-O > bonding distances shown in Table 1. On the other hand, structural transformation from ordered pyrochlore to disordered defect-fluorite structure is induced by the substitution of Sm for Zr-site, which is accompanied by the weakened interaction of < Sm-O_48*f*_ > and < Gd-O_8*b*_ > bonds (see Table 1).

**Table 1:** The bonding distances (Å) between atoms in Sm_y_Gd_2-y_Zr_2_O_7_ and Gd_2_Sm_y_Zr_2-y_O_7_ (0 ≤ y ≤ 2).

Compounds	d < Gd-O_48*f*_>	d < Gd-O_8*b*_>	d < Sm-O_48*f*_>	d < Sm-O_8*b*_>	d < Zr-O_48*f*_>
Gd_2_Zr_2_O_7_	2.478	2.263	—	—	2.083
Sm_0.5_Gd_1.5_Zr_2_O_7_	2.482	2.257	2.497	2.297	2.084
SmGdZr_2_O_7_	2.485	2.252	2.503	2.289	2.085
Sm_1.5_Gd_0.5_Zr_2_O_7_	2.495	2.245	2.506	2.286	2.086
Sm_2_Zr_2_O_7_	—	—	2.514	2.280	2.087
Gd_2_Sm_0.5_Zr_1.5_O_7_	2.482	2.280	2.253	—	2.087
Gd_2_SmZrO_7_	2.387	2.309	2.329	—	2.124
Gd_2_Sm_1.5_Zr_0.5_O_7_	2.394	2.327	2.299	—	2.096
Gd_2_Sm_2_O_7_	2.426	2.341	2.262	—	—

In the literature, it has been reported that the Madelung binding energy reduces with the increasing *x*_O48*f*_ and the thermal expansion increases with the decreasing Madelung binding energy^20^. Considering that Gd_2_Sm_y_Zr_2-y_O_7_ has larger *x*_O48*f*_ than Sm_y_Gd_2-y_Zr_2_O_7_, we thus suggest that smaller Madelung binding energy exists in the Gd_2_Sm_y_Zr_2-y_O_7_ and they probably have larger thermal expansion coefficient. Meanwhile, the disordering of oxygen ions in Gd_2_Sm_y_Zr_2-y_O_7_ may increase the phonon scattering, which will reduce the mean free path of the phonon and result in small phonon thermal conductivity.

### Elastic constants and elastic moduli of Sm_y_Gd_2-y_Zr_2_O_7_ and Gd_2_Sm_y_Zr_2-y_O_7_ (0 ≤ y ≤ 2)

In order to investigate the mechanical properties of Sm_y_Gd_2-y_Zr_2_O_7_ and Gd_2_Sm_y_Zr_2-y_O_7_ compounds, we further calculate their elastic constants based on the optimized structures. For a cubic crystal, there are three independent elastic constants, i.e. C_11_, C_12_ and C_44_, where C_11_ refers to the uniaxial deformation along the [001] direction, C_12_ is the pure shear stress at (110) crystal plane along the [110] direction, and C_44_ is a pure shear deformation on the (100) crystal plane^4^. The values of these three elastic constants for all compounds are summarized in Table 2. For Gd_2_Zr_2_O_7_, our calculated values of C_11_, C_12_ and C_44_ are 325.6, 126.3 and 94.0 GPa, respectively, which are found to be close to the results of C_11_ = 314.2 GPa, C_12_ = 126.2 GPa and C_44_ = 96.2 GPa for Sm_2_Zr_2_O_7_. It should be pointed out that in this study the Gd 4 *f* and Sm 4 *f* electrons are treated as core electrons. In order to investigate if these *f* electrons will influence the mechanical properties of Gd_2_Zr_2_O_7_ and Sm_2_Zr_2_O_7_, we further consider the Gd 4 *f* and Sm 4 *f* electrons as valence electrons and carry out LDA + U calculations, in which the effective U values are taken to be 6 eV for Gd 4 *f* and 8 eV for Sm 4 *f* electrons^4^. For Gd_2_Zr_2_O_7_, the LDA + U results of C_11_ = 316.9 GPa, C_12_ = 123.0 GPa and C_44_ = 94.7 GPa are very similar to the LDA calculations. In the case of Sm_2_Zr_2_O_7_, the calculated C_11_ = 276.9 GPa, C_12_ = 114.2 GPa, and C_44_ = 112.6 GPa are deviated from the LDA results. Further investigation shows that the Sm_2_Zr_2_O_7_ is elastically anisotropic, which is not consistent with the elastic isotropy of Sm_2_Zr_2_O_7_. Hence, the Gd 4 *f* and Sm 4 *f* electrons are frozen in our calculations and all the calculations are carried out by the LDA method. These results indicate that Gd_2_Zr_2_O_7_ and Sm_2_Zr_2_O_7_ may exhibit very similar mechanical properties, agreeing well with the experiment carried out by Shimamura *et al*.^20^. The elastic constants reported by Lan *et al*. employing GGA method are generally smaller than our LDA results, while they also found that the results of Gd_2_Zr_2_O_7_ and Sm_2_Zr_2_O_7_ are very similar to each other^36^. We find that the mechanical stability criteria, i.e., (C_11_-C_12_) > 0, C_44_ > 0, and (C_11_ + 2C_12_) > 0^1^, are satisfied for all compounds, indicating that Sm-substituted Gd_2_Zr_2_O_7_ compounds are mechanically stable.

**Table 2:** Elastic constants (C_11_, C_12_, C_44_, in GPa), bulk modulus (B, in GPa), shear modulus (G, in GPa) and Young’s modulus (E, in GPa) of Sm_y_Gd_2-y_Zr_2_O_7_ and Gd_2_Sm_y_Zr_2-y_O_7_ (0 ≤ y ≤ 2).

Compounds		C_11_	C_12_	C_44_	B	G	E
Gd_2_Zr_2_O_7_	LDA	325.6	126.3	94.0	192.7	96.2	247.5
	LDA + U	316.9	123.0	94.7	187.6	95.6	245.2
	LDA + SOC	325.6	126.6	93.7	192.9	96.0	247.0
	Exp.^23^						234
	Exp.^57^						241
	Exp.^20^				174	92	236
	Other cal.^36^	289	103	85	165	88	224
Sm_0.5_Gd_1.5_Zr_2_O_7_	LDA	320	125	95	190	96	247
SmGdZr_2_O_7_	LDA	318	125	95	189	95	245
Sm_1.5_Gd_0.5_Zr_2_O_7_	LDA	316	125	95	188	95	244
Sm_2_Zr_2_O_7_	LDA	314.2	126.2	96.2	188.8	95.3	244.7
	LDA + U	276.9	114.2	112.6	168.4	98.8	248.0
	LDA + SOC	315.3	127.4	95.9	190.0	95.1	244.5
	Exp.^23^						231
	Exp.^20^				173	91	231
	Other cal.^36^	286	107	88	169	89	226
Gd_2_Sm_0.5_Zr_1.5_O_7_	LDA	253	130	82	171	73	192
Gd_2_SmZrO_7_	LDA	209	140	68	163	52	141
Gd_2_Sm_1.5_Zr_0.5_O_7_	LDA	190	139	59	156	42	116
Gd_2_Sm_2_O_7_	LDA	165	144	61	151	31	87

**Figure 3:**
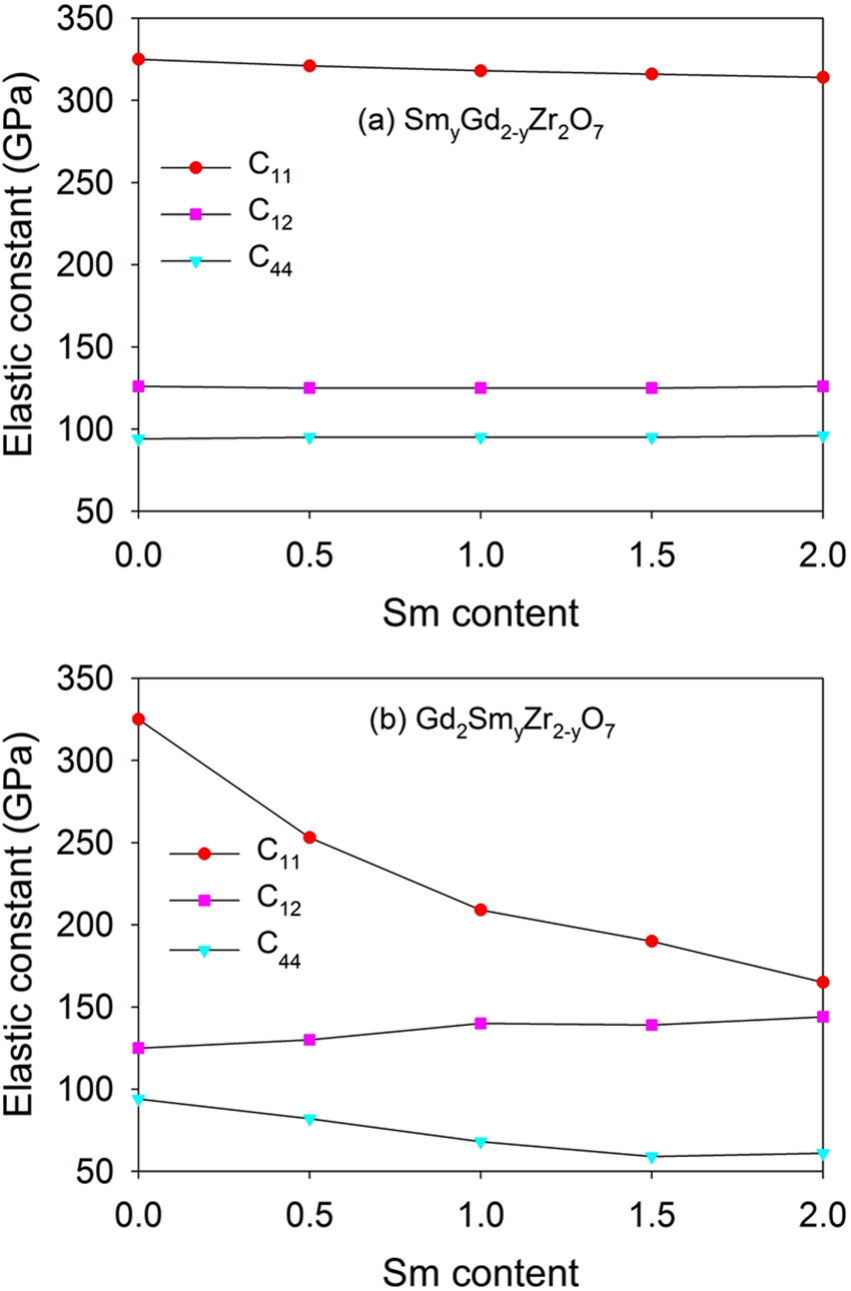
Variation of elastic constants (C_11_, C_12_ and C_44_) for (**a**) Sm_y_Gd_2-y_Zr_2_O_7_ and (**b**) Gd_2_Sm_y_Zr_2-y_O_7_ (0 ≤ y ≤ 2) with Sm content.

The calculated elastic constants for both Sm_y_Gd_2-y_Zr_2_O_7_ and Gd_2_Sm_y_Zr_2-y_O_7_ (0 ≤ y ≤ 2) as a function of Sm content are plotted in Fig. 3. It is noted that Sm substitution for Gd-site results in very small changes in the elastic constants and the mixed Gd_2_Zr_2_O_7_-Sm_2_Zr_2_O_7_ phases have similar values with the pure states. As the Sm substitutes for Zr-site, the values of C_11_ are strikingly decreased with the increasing Sm content, implying that the compositions with high content of Sm are more likely to undergo the uniaxial deformation along the [001] direction. On the other hand, the C_12_ and C_44_ slightly increase and decrease with the Sm incorporation, respectively. Generally, the changes in the elastic constants of Gd_2_Sm_y_Zr_2-y_O_7_ are more significant than those of Sm_y_Gd_2-y_Zr_2_O_7_, meaning that the effects of Sm incorporation into Gd-site on the mechanical properties of Gd_2_Zr_2_O_7_ are nearly negligible whereas Sm incorporation into Zr-site has remarkable influence. This may be mainly due to the fact that Gd^3+^ and Sm^3+^ have similar mass (Gd^3+^: 157.25 amu; Sm^3+^: 150.3 amu) and cation size (Gd^3+^: 1.053 Å; Sm^3+^: 1.079 Å) and Sm substitution for Gd-site affects the structural properties of Gd_2_Zr_2_O_7_ slightly, while the mass and radius for Gd^3+^ are largely different from the mass of 91.22 amu and the radius of 0.72 Å for Zr^4+^ ^19, 27^ and an order-disorder phase transition has occurred in Gd_2_Sm_y_Zr_2-y_O_7_.

**Figure 4:**
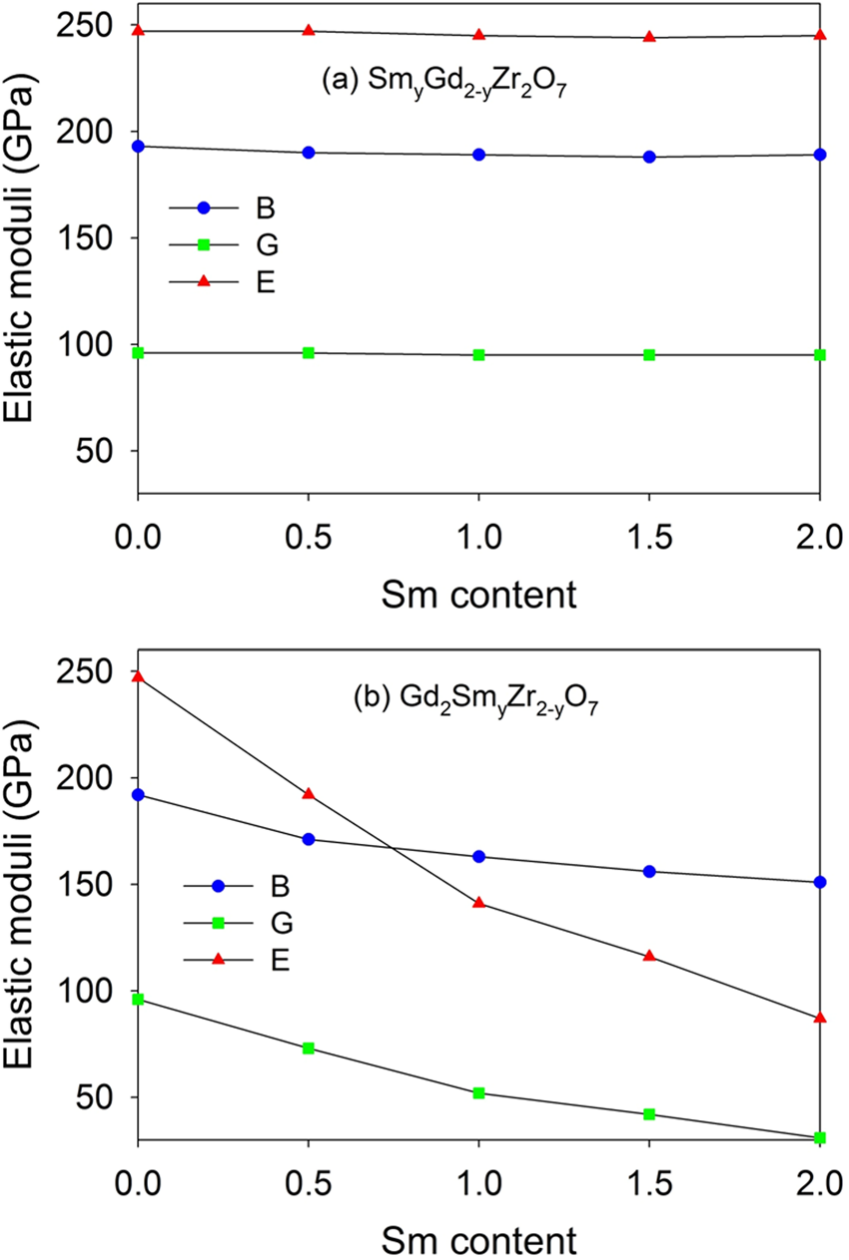
Variation of elastic moduli for (**a**) Sm_y_Gd_2-y_Zr_2_O_7_ and (**b**) Gd_2_Sm_y_Zr_2-y_O_7_ (0 ≤ y ≤ 2) as a function of Sm content. B: bulk modulus; G: shear modulus; E: Young’s modulus.

Based on the three elastic constants, the bulk modulus (B), Young’s modulus (E) and shear modulus (G) can be deduced under Voigt-Reuss-Hill (VRH) approximation^37^, i.e., B = (C_11_ + 2C_12_)/3, E = 9BG/(3B + G), G = ((C_11_-C_12_ + 3C_44_)/5 + 5(C_11_-C_12_)C_44_/(4C_44_ + 3(C_11_-C_12_)))/2^1, 38–40^. The values of bulk modulus, Young’s modulus and shear modulus are shown in Table 2, together with available experimental and theoretical results in the literature^20, 36^. As compared with the experimental measurement, our calculated elastic moduli for Gd_2_Zr_2_O_7_ and Sm_2_Zr_2_O_7_ are overestimated slightly. This may be resulted from the employed LDA method, which generally underestimates the lattice constant whereas overestimates the mechanical modulus^41^. Besides, the defects and impurities in the sample experimentally may also lead to the underestimated values of the B and G^41^. Variation of the elastic moduli for Sm_y_Gd_2-y_Zr_2_O_7_ and Gd_2_Sm_y_Zr_2-y_O_7_ with the Sm content is illustrated in Fig. 4. As expected, the elastic moduli for Sm_y_Gd_2-y_Zr_2_O_7_ vary slightly with the Sm content since the elastic constants for all compositions are very similar to each other. Different from the case of Sm_y_Gd_2-y_Zr_2_O_7_, the bulk modulus, Young’s modulus and shear modulus for Gd_2_Sm_y_Zr_2-y_O_7_ all decrease sharply with the increasing Sm content, especially the Young’s modulus. Consequently, the Gd_2_Sm_2_O_7_ has the minimum Young’s modulus of 87 GPa, minimum shear modulus of 31 GPa and minimum bulk modulus of 151 GPa. These results indicate that the Gd_2_Sm_2_O_7_ has good compliance^2^ due to the lowest bulk modulus and the lowest Young’s modulus, which will produce relatively smaller residual stresses in the coating system under the severe conditions and result in better thermo-mechanical stability^23^.

### Elastic anisotropy, ductility and Debye temperature of Sm_y_Gd_2-y_Zr_2_O_7_ and Gd_2_Sm_y_Zr_2-y_O_7_ (0 ≤ y ≤ 2)

The elastic anisotropy, which is correlated with the possibility of the appearance of microcracks, is an important mechanical property of materials^42, 43^. For a crystal, the elastic anisotropy of materials can be evaluated by the A^U^ (universal elastic anisotropy index), which can be calculated by A^U^ = 5 G^V^/G^R^ + B^V^/B^R^-6^44^, with V and R representing the Voigt and Reuss approximation, respectively^38, 40^. The A^U^ value of zero refers to isotropic mechanical properties, otherwise defines the anisotropy^44^. The calculated results are shown in Table 3. Obviously, the values of A^U^ are very close to zero in the case of Sm_y_Gd_2-y_Zr_2_O_7_ (0 ≤ y ≤ 2), which indicates that all compounds are of elastic isotropy. However, the crystals show strong anisotropy in the Gd_2_Sm_y_Zr_2-y_O_7_ (0.5 ≤ y ≤ 2) system, as the A^U^ values have large deviation from zero and they increase with the increasing Sm content. The sharp increase from 0.920 for Gd_2_Sm_1.5_Zr_0.5_O_7_ to 4.738 for Gd_2_Sm_2_O_7_ may be caused by the pyrochlore-to-defect fluorite structural transition.

**Table 3:** Pugh’s indicator (G/B), elastic anisotropy index (A^U^), sound wave velocity (*v*_*m*_, in m/s), Debye temperature (*Θ*, in K) and Poisson’s ratio (σ) of Sm_y_Gd_2-y_Zr_2_O_7_ and Gd_2_Sm_y_Zr_2-y_O_7_ (0 ≤ y ≤ 2).

Compounds		G/B	A^U^	*v*_*m*_	*Θ*	σ
Gd_2_Zr_2_O_7_	Our Cal.	0.499	0.00420	4833.5	612.9	0.286
	Exp.^48^					0.276
	Exp.^20^				513.3	0.274
	Other cal.^36^					0.273
Sm_0.5_Gd_1.5_Zr_2_O_7_		0.505	0.00085	4855.2	614.4	0.284
SmGdZr_2_O_7_		0.504	0.00042	4864.9	614.5	0.284
Sm_1.5_Gd_0.5_Zr_2_O_7_		0.504	0.00009	4880.8	615.3	0.284
Sm_2_Zr_2_O_7_	Our Cal.	0.505	0.00063	4918.3	618.8	0.284
	Exp.^48^					0.277
	Exp.^20^				513.3	0.278
	Other cal.^36^					0.274
Gd_2_Sm_0.5_Zr_1.5_O_7_	Our Cal.	0.428	0.097	4220.6	530.5	0.313
Gd_2_SmZrO_7_	Our Cal.	0.320	0.566	3583.7	447.4	0.356
Gd_2_Sm_1.5_Zr_0.5_O_7_	Our Cal.	0.270	0.920	3230.2	399.5	0.376
Gd_2_Sm_2_O_7_	Our Cal.	0.205	4.738	2786.4	341.7	0.404

Another important mechanical property of materials is the ductility, which is often evaluated by the Pugh’s indicator (G/B ratio)^45^. The Pugh’s indicator of 0.5 is a boundary of brittleness or ductility, i.e., if G/B > 0.5, the material tends to be brittle; otherwise, the material is ductile^46^. The calculated Pugh’s indicators are presented in Table 3. For the compounds of Sm_y_Gd_2-y_Zr_2_O_7_, the values are close to 0.5, and are located within the range of 0.499 to 0.505. As for Gd_2_Sm_y_Zr_2-y_O_7_ (y = 0.5, 1, 1.5, 2), the G/B values of 0.205–0.428 are much smaller. Obviously, the Gd_2_Sm_y_Zr_2-y_O_7_ compositions have better ductility than the Sm_y_Gd_2-y_Zr_2_O_7_ compounds. The Poisson’s ratio (σ) can also be used to evaluate the relative ductility of materials. Generally, the σ values are close to 0.1 and 0.33 for brittle covalent material and ductile metallic material, respectively^46, 47^. The σ values are 0.286 and 0.284 for Gd_2_Zr_2_O_7_ and Sm_2_Zr_2_O_7_, respectively, which are comparable with the experimental data of 0.274–0.276 for Gd_2_Zr_2_O_7_ and 0.277–0.278 for Sm_2_Zr_2_O_7_^20, 48^. As shown in Table 3, the Poisson’s ratio are ~0.285 for Sm_y_Gd_2-y_Zr_2_O_7_ and vary from 0.313 to 0.404 for Gd_2_Sm_y_Zr_2-y_O_7_, meaning that the latter compositions are more ductile, which is consistent with the results obtained from the Pugh’s indicator.

In this study, the Debye temperature that is related to the hardness and thermal expansion coefficient of materials^20, 49^ is also estimated for Sm-contained Gd_2_Zr_2_O_7_ by $\Theta =\frac{h}{{k}_{B}}{[\frac{3n}{4\pi }(\frac{{N}_{A}\rho }{M})]}^{\frac{1}{3}}{v}_{m}$. Here, *h* is the Planck’s constant, *k*_*B*_ is the Boltzmann’s constant, *n* is the number of atoms in molecular, *N*_*A*_ is the Avogadro’s constant, *ρ* is the density, *M* is the molecular mass and *v*_*m*_ is the sound wave velocity. The *v*_*m*_ can be deduced by ${v}_{m}={(\frac{3{({v}_{t}{v}_{l})}^{3}}{2{v}_{t}^{3}+{v}_{l}^{3}})}^{\frac{1}{3}}$, where ${v}_{l}={(\frac{B+4G/3}{\rho })}^{\frac{1}{2}}$ is the longitudinal sound velocity and ${v}_{t}={(\frac{G}{\rho })}^{\frac{1}{2}}$ is the transverse sound velocity^41^. As one can see from Table 3, Sm substitution for Gd-site has slight effects on the Debye temperature of Gd_2_Zr_2_O_7_ and all the compositions have very similar results. However, as Sm substitutes for the Zr-site, the Debye temperature decreases considerably with the increasing Sm content. The Gd_2_Sm_2_O_7_ has the lowest Debye temperature of 341.7 K, which is about 44.3% lower than that of Gd_2_Zr_2_O_7_. These results suggest that Sm incorporation into Zr-site causes weaker interaction of chemical bonds and the Gd_2_Sm_y_Zr_2-y_O_7_ with high content of Sm will have much larger thermal expansion coefficient than Gd_2_Zr_2_O_7_.

### Electronic structure of Sm_y_Gd_2-y_Zr_2_O_7_ and Gd_2_Sm_y_Zr_2-y_O_7_ (0 ≤ y ≤ 2)

**Figure 5:**
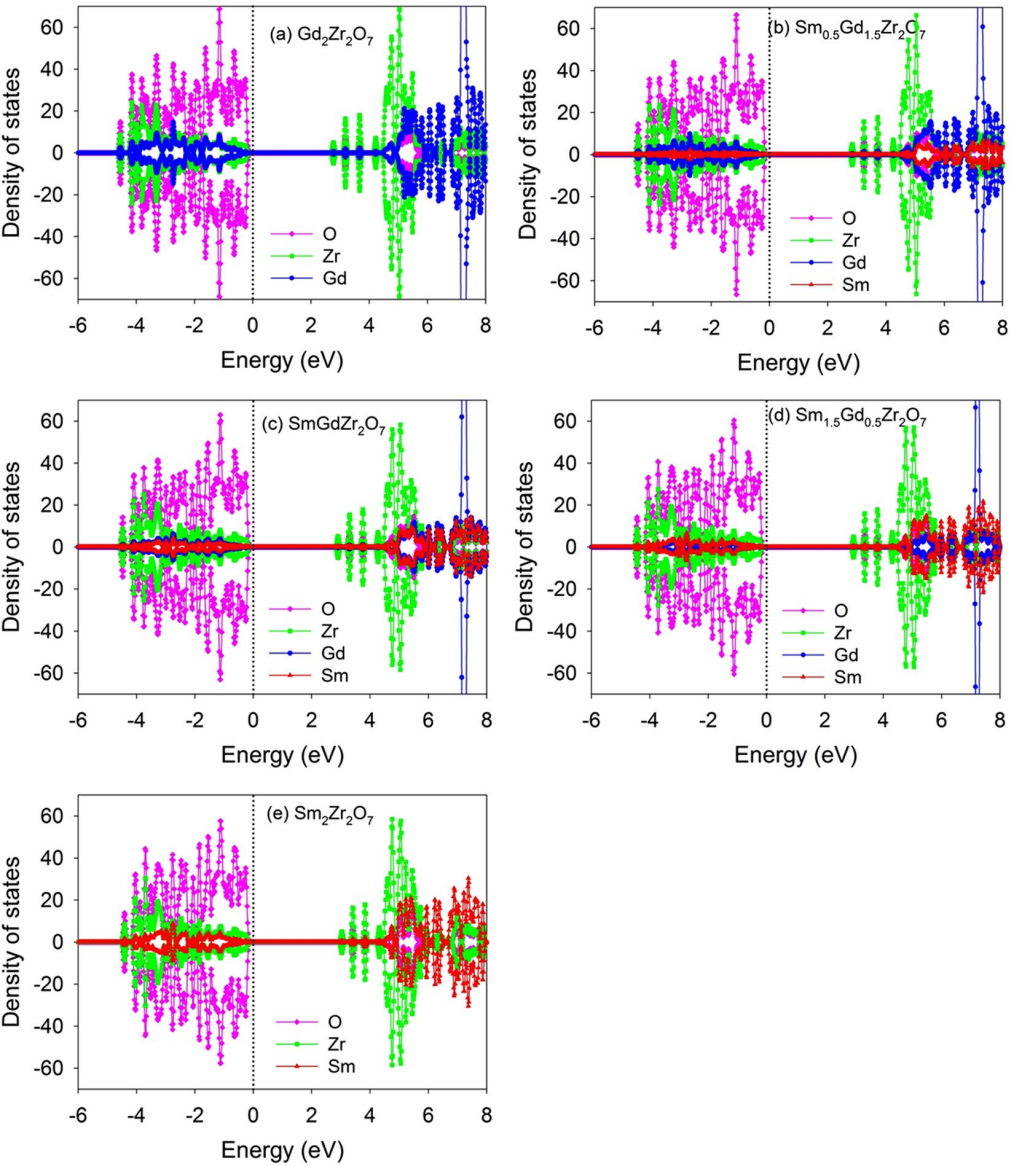
Projected density of state distribution for Sm_y_Gd_2-y_Zr_2_O_7_ (y = 0, 0.5, 1, 1.5, 2). The Fermi level is located at 0 eV.

**Figure 6:**
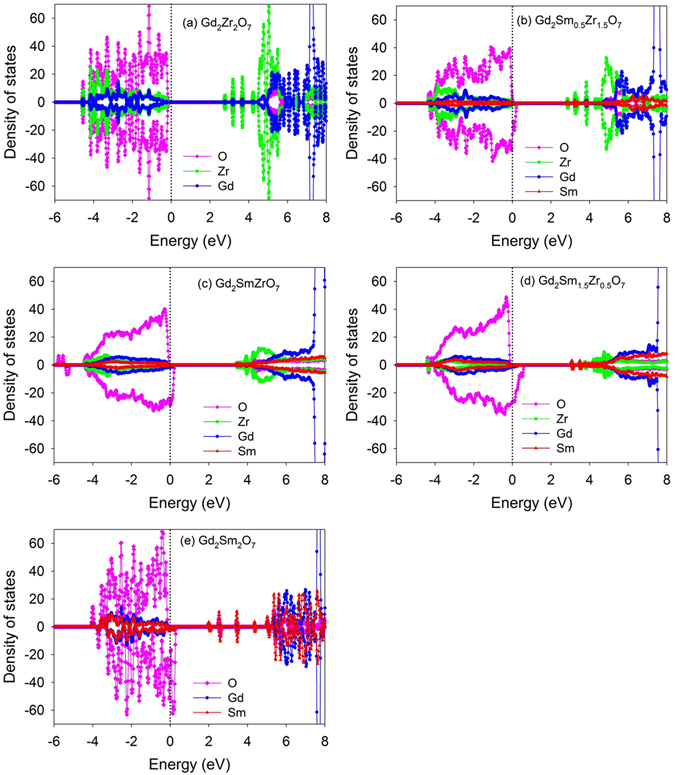
Projected density of state distribution for Gd_2_Sm_y_Zr_2-y_O_7_ (y = 0, 0.5, 1, 1.5, 2). The Fermi level is located at 0 eV.

**Figure 7:**
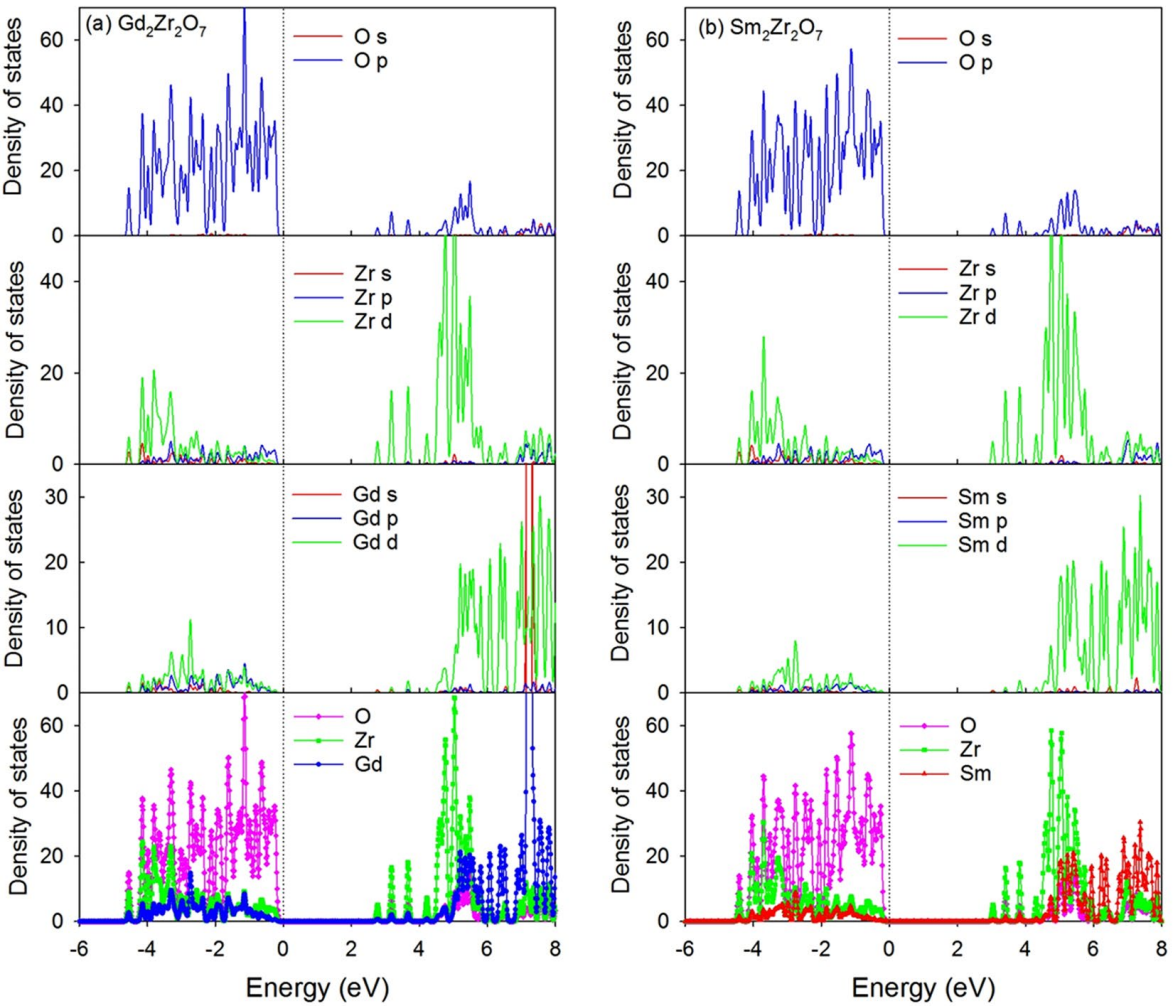
Projected density of state distribution for Gd_2_Zr_2_O_7_ and Sm_2_Zr_2_O_7_. The Fermi level is located at 0 eV.

**Figure 8:**
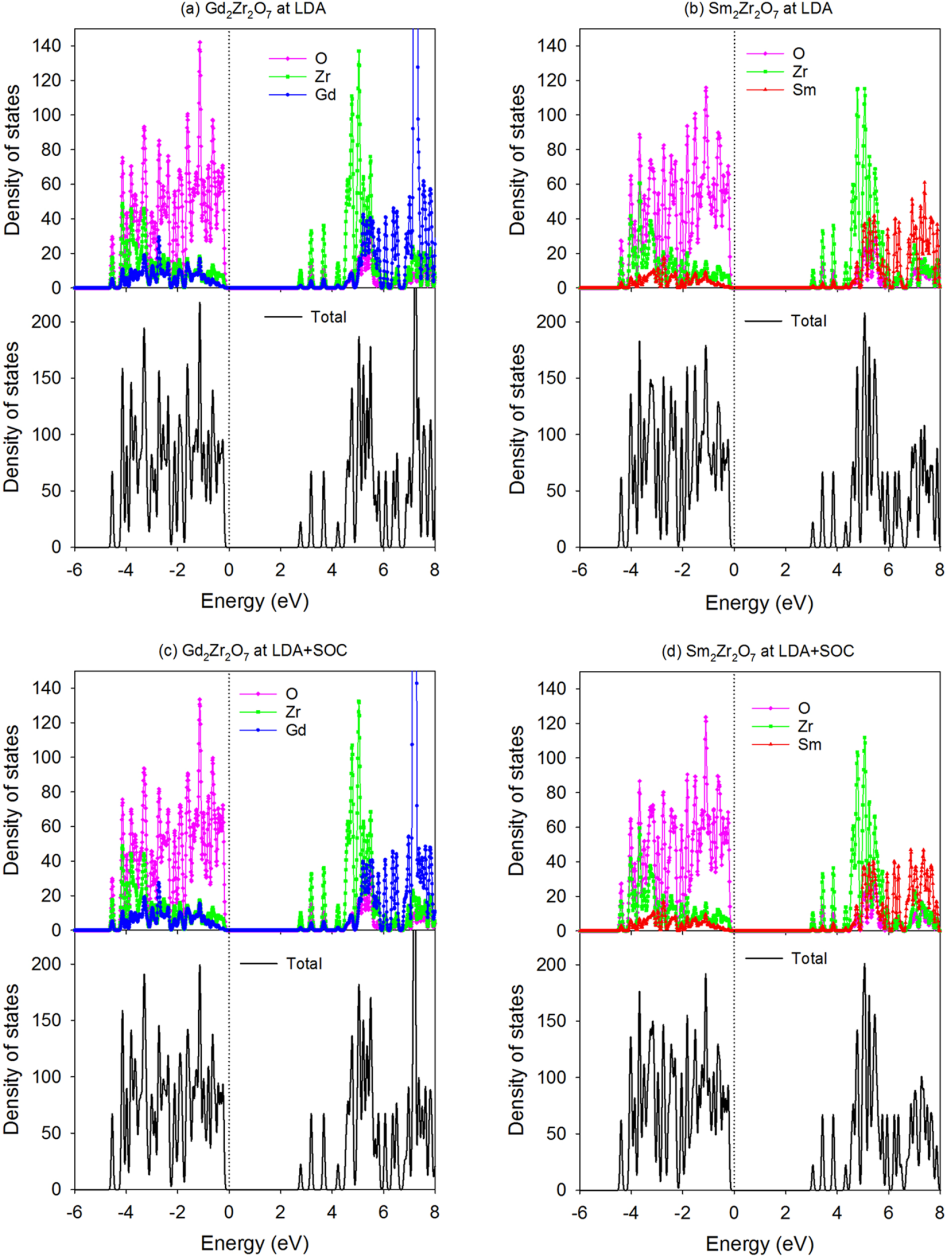
Projected and total density of state distribution for Gd_2_Zr_2_O_7_ and Sm_2_Zr_2_O_7_ obtained by the LDA and LDA + SOC methods. The Fermi level is located at 0 eV.

In order to investigate how Sm incorporation influences the electronic structure of Gd_2_Zr_2_O_7_, the atomic projected density of state (DOS) distribution of Sm_y_Gd_2-y_Zr_2_O_7_ and Gd_2_Sm_y_Zr_2-y_O_7_ are analyzed and plotted in Fig. 5 and Fig. 6, respectively. In Fig. 7a and b, the orbital projected DOS for Gd_2_Zr_2_O_7_ and Sm_2_Zr_2_O_7_ are also presented. For Gd_2_Zr_2_O_7_, the valence band maximum (VBM) are mainly contributed by O 2*p* states hybridized with Zr 4*d*, Gd 5*d* and Gd 5*p* orbitals, and the conduction band minimum (CBM) are mainly contributed by Zr 4*d* states and O 2*p* states. The obtained band gap of 2.55 eV is comparable with the value of 2.71 eV reported by Wang *et al*.^50^. For Sm_2_Zr_2_O_7_, the DOS distribution is similar to that of Gd_2_Zr_2_O_7_, i.e., the VBM are mainly contributed by O 2*p* states hybridized with Zr 4*d*, Sm 5*d* and Sm 5*p* states, and the CBM are mainly dominated by Zr 4*d* states and O 2*p* states. Considering that Sm and Gd are heavy atoms and the spin-orbit coupling (SOC) may affect the band gap and electronic structure of the investigated systems, we further calculate the density of state distribution of Gd_2_Zr_2_O_7_ and Sm_2_Zr_2_O_7_ employing the LDA + SOC method. A comparison of LDA and LDA + SOC results for both compounds is illustrated in Fig. 8. For Gd_2_Zr_2_O_7_, the band gap value of 2.64 eV by LDA + SOC is close to the value of 2.55 eV by LDA method. In the case of Sm_2_Zr_2_O_7_, the band gap values are 2.9 eV and 2.83 eV for LDA + SOC and LDA calculations, respectively. As shown in Fig. 8, the atomic projected and total DOS obtained by LDA with and without spin-orbit coupling exhibit very similar characters for both compounds. These results suggest that the spin-orbit coupling has slight effects on our results and such effects are thus not considered for the mixed states. When Sm substitutes for Gd-site, the Sm 5*d* orbitals also contribute to the VBM and interacts with the oxygen. Meanwhile, the insulating character of Gd_2_Zr_2_O_7_ is kept and the band gap is broadened with the increasing Sm content, i.e., 2.62, 2.68, 2.76 and 2.83 eV for Sm_0.5_Gd_1.5_Zr_2_O_7_, SmGdZr_2_O_7_, Sm_1.5_Gd_0.5_Zr_2_O_7_ and Sm_2_Zr_2_O_7_, respectively. In addition, the hybridization of O 2*p* and Zr 4*d* is slightly affected by the incorporation of Sm. As for Gd_2_Sm_y_Zr_2-y_O_7_, the hybridization of O 2*p* states with Zr 4*d*, Gd 5*d* and Sm 5*d* orbitals also dominates the VBM. It is interesting to find that Sm incorporation causes electrons distributing on the fermi levels and the number of electrons increases with the increasing Sm content. This results in an insulating-to-metallic transition and the Gd_2_Sm_y_Zr_2-y_O_7_ has much stronger electronic conductivity than the pure state. In the meantime, the Gd_2_Zr_2_O_7_ and Sm_y_Gd_2-y_Zr_2_O_7_ do not have any magnetism, whereas the Gd_2_Sm_y_Zr_2-y_O_7_ compositions exhibit strong ferromagnetic states due to the different charge states of Sm and Zr.

Obviously, Sm incorporation into Gd-site and Zr-site of Gd_2_Zr_2_O_7_ causes very different electronic structures, i.e., the Sm_y_Gd_2-y_Zr_2_O_7_ and Gd_2_Sm_y_Zr_2-y_O_7_ are mainly of insulating and metallic characters, respectively. These results indicate that the thermal conductivity of Sm_y_Gd_2-y_Zr_2_O_7_ are mainly contributed by phonons, while both phonons and electrons contribute to the thermal conductivity of Gd_2_Sm_y_Zr_2-y_O_7_.

## Summary

In this work, a systematic study based on the DFT method is carried out to investigate the effects of Sm substitution for Gd-site and Zr-site in Gd_2_Zr_2_O_7_ on its structural stability, mechanical properties, Debye temperature and electronic structures. It is shown that the Sm_y_Gd_2-y_Zr_2_O_7_ compositions keep the pyrochlore structure and their lattice parameters follow well the Vegard’s law, indicative of the formation of Gd_2_Zr_2_O_7_-Sm_2_Zr_2_O_7_ solid solution. On the other hand, Sm substitution for Zr-site influences the structure significantly and a pyrochlore-to-defect fluorite structural transition occurs. The Sm_y_Gd_2-y_Zr_2_O_7_ compositions are of elastic isotropy and their elastic moduli, ductility, Debye temperature and band gap vary slightly with the Sm content. However, the Gd_2_Sm_y_Zr_2-y_O_7_ compounds show strong elastic anisotropy and their bulk, Young’s and shear moduli all decrease sharply with the increasing Sm content. Consequently, the Gd_2_Sm_2_O_7_ has the minimum Young’s modulus of 87 GPa, minimum shear modulus of 31 GPa and minimum bulk modulus of 151 GPa. Meanwhile, both the Pugh’s indicator and Poisson’s ratio suggested that the Gd_2_Sm_y_Zr_2-y_O_7_ have better ductility than the Sm_y_Gd_2-y_Zr_2_O_7_. As the Sm substitutes for Zr-site, the Debye temperature decreases considerably with the increasing Sm content and the Debye temperature of 341.7 K for Gd_2_Sm_2_O_7_ is about 44.3% lower than that of Gd_2_Zr_2_O_7_. In addition, the insulating character of Gd_2_Zr_2_O_7_ is kept in the system of Sm_y_Gd_2-y_Zr_2_O_7_, while the Gd_2_Sm_y_Zr_2-y_O_7_ compositions exhibit metallic characters. Our calculations demonstrate that substituting Sm for Zr-site is an effective approach to tailor the mechanical and thermal properties of Gd_2_Zr_2_O_7_.

## Methods

**Figure 9:**
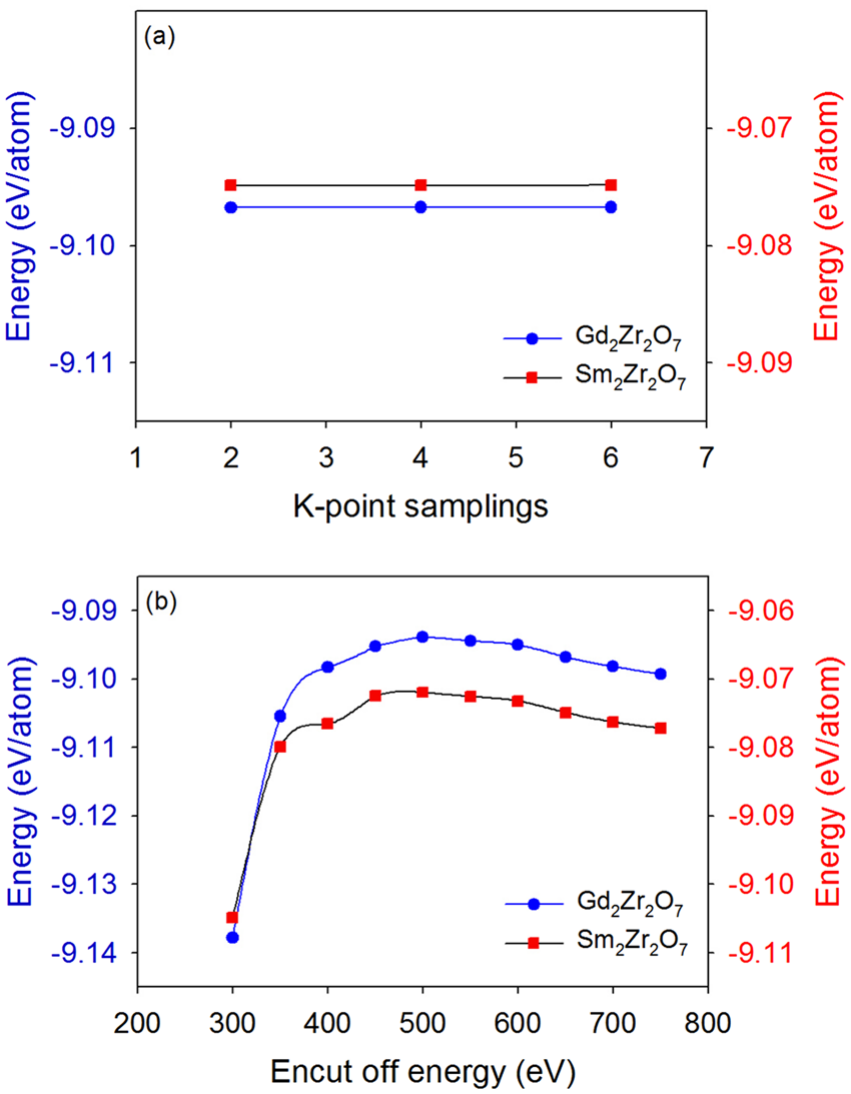
Variation of the total energy of Gd_2_Zr_2_O_7_ and Sm_2_Zr_2_O_7_ with (**a**) k-point sampling (the cutoff energy is fixed at 650 eV) and (**b**) cutoff energy (the k-point sampling is fixed at 2 × 2 × 2).

In this work, first-principles total energy calculations within the DFT framework are carried out. All calculations are performed with the Vienna Ab-initio Simulation Package (VASP)^51, 52^. The interaction between electrons and ions is described by the projector augmented wave method^52, 53^. All computations are based on a supercell containing 88 atoms. The convergence criteria for total energies and forces are 10^−6^ eV and 10^−6^ eV/Å, respectively. The structural relaxation is carried out at variable volume. In order to determine the values of cutoff energy and k-point sampling, a series of test calculation has been carried out. Figure 9 shows the variation of total energy of Gd_2_Zr_2_O_7_ and Sm_2_Zr_2_O_7_ with cutoff energy and k-point sampling, which leads to our calculation being performed with a 2 × 2 × 2 Monkhorst-Pack k-mesh for Brillouin-zone integrations and a cutoff energy of 600 eV for plane wave. For Sm_y_Gd_2-y_Zr_2_O_7_ and Gd_2_Sm_y_Zr_2-y_O_7_ (y = 0.5, 1, 1.5), the structure models are constructed by the special quasirandom structure approach^54–56^.
